# Determination of the Effect of Wastewater on the Biological Activity of Mixtures of Fluoxetine and Its Metabolite Norfluoxetine with Nalidixic and Caffeic Acids with Use of *E. coli* Microbial Bioindicator Strains

**DOI:** 10.3390/ma16093600

**Published:** 2023-05-08

**Authors:** Marzena Matejczyk, Piotr Ofman, Józefa Wiater, Renata Świsłocka, Paweł Kondzior, Włodzimierz Lewandowski

**Affiliations:** 1 Faculty of Civil Engineering and Environmental Sciences, Department of Chemistry, Biology and Biotechnology, Bialystok University of Technology, Wiejska 45E Street, 15-351 Bialystok, Poland; 2 Department of Technology in Environmental Engineering, Bialystok University of Technology, Wiejska 45E Street, 15-351 Bialystok, Poland; 3 Department of Agri-Food Engineering and Environmental Management, Bialystok University of Technology, Wiejska 45E Street, 15-351 Bialystok, Poland

**Keywords:** toxicity, fluoxetine, nalidixic acid, caffeic acid, wastewaters, microbial bioindicator

## Abstract

In the present work, the conducted research concerned the determination of the toxicity and oxidative stress generation of the antidepressant fluoxetine (FLU), its metabolite nor-fluoxetine (Nor-FLU), the antibiotic nalidixic acid (NA), caffeic acid (CA) and their mixtures in three different environments: microbial medium (MM), raw wastewaters (RW) and treated wastewaters (TW). We evaluated the following parameters: *E. coli* cell viability, toxicity and protein damage, *sodA* promoter induction and ROS generation. It was found that FLU, Nor-FLU, NA, CA and their mixtures are toxic and they have the potency to generate oxidative stress in *E. coli* strains. We also detected that the wastewater, in comparison to the microbial medium, had an influence on the toxic activity and oxidative stress synthesis of the tested chemicals and their mixtures. Regardless of the environment under study, the strongest toxic activity and oxidative stress generation were detected after bacterial incubation with NA at a concentration of 1 mg/dm^3^ and the mixture of FLU (1 mg/dm^3^) with Nor-FLU (0.1 mg/dm^3^) and with NA (0.1 mg/dm^3^). The ROS synthesis and *sodA* promoter induction suggest that, in the case of the examined compounds and their mixtures, oxidative stress is the mechanism of toxicity. The analysis of the types of interactions among the substances constituting the mixtures in the wastewater revealed synergism, potentiation and antagonism.

## 1. Introduction

Fluoxetine (FLU) is one of the most frequently prescribed antidepressants in the world. FLU belongs to the group of serotonin reuptake inhibitors—SSRIs. FLU is one of the world’s best-selling prescription antidepressants [1]. These drugs are prescribed for the treatment of mental disorders such as depression, obsessive–compulsive disorder, phobias and anxiety [2,3]. Serotonin reuptake inhibition leads to an increase in the concentration of this neurotransmitter in the brain [2,3,4]. FLU in the human body, by N-demethylation and with the participation of several CYP450 enzymes, including CYP2D6, CYP2C19, CYP2C9 and CYP3A5, is metabolized to the active metabolite norfluoxetine (Nor-FLU) [3,5]. In recent years, the consumption of SSRIs has increased significantly [3,4]. The consequence of the increase in the consumption of SSRIs is the increase in the detectability of these drugs and their metabolites in the environment. So far, the presence of antidepressants, including FLU and their metabolites, has been repeatedly documented in wastewater in a wide range of concentrations from 0.15 to 32,228 ng/dm^3^, in surface waters in concentrations from 0.5 up to 8000 ng/dm^3^ and in tap water, from 0.5 to 1400 ng/dm^3^. In acute toxicity tests conducted on *Xenopus laevis*, the LC_50_ value after 96 h exposure for FLU was 7.5 mg/dm^3^ [3,6,7,8]. Drugs belonging to the group of SSRIs, including FLU, are characterized by a relatively high bioaccumulation capacity at concentrations of ng/g in aquatic organisms such as fish and molluscs. In addition to these drugs, their metabolites Nor-FLU (norfluoxetine) and Nor-SER (norsertraline) have also been detected in the tissues of aquatic organisms, including the brain, liver and muscles [6,7]. The toxic activity of FLU was demonstrated in tests on microorganisms such as *Pseudomonas aeruginosa*, *Staphylococcus aureus*, *Escherichia coli*, *Acinetobacter baumannii* and higher organisms [8]. In toxicological tests carried out on fish and molluscs, it has been documented that SSRIs, including FLUs used at the concentration found in the environment, cause disturbances in many anatomical and physiological processes [9,10]. In toxicity tests performed on fish, FLU was found to affect their sex hormones and modulate the activity of genes involved in reproductive functions [11]. Gil-Ad et al. [12] found in their work that FLU inhibits the proliferation of prostate cancer cells and induces apoptosis in Burkitt’s lymphoma cells. In the work of Nałęcz-Jawecki et al. [13], the toxicity and bioaccumulation potential of FLU in the test on the protozoan *Spirostomum ambiguum* were documented.

In recent years, we have seen a continuous increase in the use of antibiotics around the world, which in turn leads to the release of more and more of these drugs into the environment. Antibiotics are often detected in wastewater, surface waters, soil, plants, animal tissues and products and in human milk at concentrations ranging from ng/L to µg/L [14]. Nalidixic acid (NA), an antibiotic that belongs to the quinolones, is widely used in human and veterinary medicine. Nalidixic acid selectively and reversibly inhibits DNA synthesis in bacteria [15]. NA is frequently identified in surface water and wastewater. The observed concentrations of NA in WWTP influents were within the range of 6–50 ng/L. In surface water, this drug is detected at concentrations of 10 ng/L to 2 µg/L [15,16]. Many studies have demonstrated the toxicity of NA against mainly Gram-negative bacteria and fungi, as well as mice and rats. Antibiotic exposure might also affect human health. In addition, the release of antibiotics into the environment contributes to the spread of the phenomenon of antibiotic resistance in microorganisms [15,17].

In this work, we selected caffeic acid (CA) as a substance potentially interacting with FLU. Caffeic acid (CA), as well as other natural plant phenolic compounds, occurs in coffee beans. CA is also found in fruits, vegetables, wine and olive oil. CA, in combination with other products of natural origin, e.g., propolis, is used in human medicine. CA is characterized by multidirectional biological activity. To date, the antibacterial, antiviral, antioxidant and antitumor activities of caffeic acid and its derivatives have been found [18,19]. Moreover, in recent years, CA has been used to promote hematopoiesis and hemostasis and to treat various causes of thrombocytopenia and leukocytopenia in China [20]. CA, due to its popularity in human diets, leaks into the environment (into wastewater and surface waters), where it can interact with FLU and its metabolite, Nor-FLU. The most common concentrations of CA in wastewater are µg/L. Wastewater from the wine industry is characterized by a high concentration of phenolic compounds, including caffeic acid, which may cause environmental pollution and risks to human health. CA is refractory to conventional wastewater treatments. CA exhibits high toxicity and represents a risk to human health when exposed at concentrations of 1.3 g/L [19]. In acute toxicity tests conducted on rats and mice, the LC_50_ reaches values of 1160 mg/kg and 572 mg/kg, respectively [19].

The rapid increase in the detection of drugs and their metabolites in the environment in recent years creates the need to conduct more studies on the toxicity of pharmaceuticals, their metabolites and their mixtures, and also on other chemical compounds present in wastewater and surface water. Pharmaceuticals, metabolites and their mixtures affect the composition of the activated sludge microflora and the biodiversity of activated sludge microorganisms. Pharmaceuticals change the metabolism of activated sludge microorganisms and the size of flocs, which can lead to disturbances in their proper functioning and a decrease in the biodegradation efficiency of pollutants [21,22,23]. Additionally, it is important to know to what extent the wastewater environment affects the toxicity of drugs, their metabolites and their mixtures.

In this paper, we present the toxicity of FLU, Nor-FLU, NA and CA and their mixtures in microbial medium (MM), raw wastewater (RW) and treated wastewater (TW). We studied the toxicity, protein damage and oxidative stress generation of tested chemicals and their mixtures in *E. coli* strains. We also performed an analysis of the influence of RW and TW in comparison to the MM on the tested parameters. The toxicity of FLU, Nor-FLU, NA, CA and their mixtures was determined on the basis of *E. coli* viability inhibition, *grpE* promoter induction in the *E. coli* RFM443 *grpE:luxCDABE* strain and bioluminescence decrease in the *E. coli* RFM443 *lac:luxCDABE* strain. The level of oxidative stress generation was evaluated on the basis of *sodA* promoter induction in *E. coli* SM345 *sodA:luxCDABE* and ROS synthesis. Strains of bacteria with the *lux* reporter gene, used in this research were successfully applied to the detection of toxicity and oxidative stress generation in different environmental pollution analyses of wastewaters in earlier works [24,25,26,27,28].

The model object in the presented study was *E. coli* strains. Due to their well-known biochemistry, genetics and physiology, *E. coli* are a frequent subject of toxicological research. *E. coli* is detected in the environment, wastewater, soil, and surface waters, where the microorganisms may potentially be exposed to chemical pollutants, including SSRIs and antibiotics. Moreover, earlier scientific works have repeatedly documented that FLU, NA and CA also have antibacterial activity against *E. coli* [8,15,19].

The present work is part of a broader topic (grant NCN No. 2018/29/NZ9/01997) that aims to investigate the: (a) preservative properties of selected phenolic acids in model systems and physiological fluids in the natural environment, (b) relationship between molecular structure and biological activity, (c) synergistic effect and (d) biodegradation and toxicity of selected phenolic compounds and their metabolites.

## 2. Materials and Methods

### 2.1. Chemical Preparation

The FLU, Nor-FLU, NA and CA were purchased from Sigma-Aldrich. All chemicals were dissolved in dimethyl sulfoxide (DMSO). To reduce the impact of DMSO on the bacterial culture, prepared solutions of tested compounds in DMSO were diluted in the tested medium at a ratio of 1:9 (1 mL DMSO and 9 mL medium). The FLU and NA were added to the *E. coli* cultures for a final concentration of 0.01, 0.1 and 1 mg/dm^3^; the CA had a concentration of 1 mg/dm^3^. The mixtures of FLU with NA and CA have been prepared in the following proportions: (a) 0.01, 0.1 and 1 mg/dm^3^ of FLU plus 0.1 mg/dm^3^ NA or Nor-FLU, (b) 0.01, 0.1 and 1 mg/dm^3^ of FLU plus 1 mg/dm^3^ CA. In choosing the concentrations of the drugs tested in this work, we were guided by the concentrations of antidepressants and antibiotics found in wastewater around the world, and we also tested higher concentrations. In hospital wastewater, concentrations of antibiotics detected reach above 100 µg/L [4] and antidepressants above 32 µg/L [3,4,7]. In the mixtures tested in our work, we used concentrations of NA (0.1 mg/dm^3^) and the metabolite Nor-FLU (0.1 mg/dm^3^), which correspond to the concentrations of drugs found in wastewater.

### 2.2. E. coli Strains Used in the Study

For toxicity assessment, we used an *E. coli* (ATCC 25922) strain obtained from the American Type Culture Collection (Manassas, VA, USA). Moreover, for toxicity and protein damage determination, we applied *E. coli* RFM443 strains with a transcriptional fusion of *grpE* or *lac* promoters with the *luxCDABE* reporter gene [25]. In the oxidative stress test, we used an *E. coli* SM342 *sodA:luxCDABE* strain with superoxide stress-responsive *sodA* promoter. Genetic plasmid fusion with the *sodA* promoter is commonly used in oxidative stress tests [24,26,27,28,29]. The work of *E. coli luxCDABE* bioindicator strains consists of the production of bioluminescence, which is the result of bacterial luciferase enzyme activity [24,29]. The *E. coli* strains used in this study are presented in Table 1.

### 2.3. Wastewater Preparation

The municipal wastewater samples were taken over the years 2020–2021 from the Rajgród WWTPs. The raw wastewater (RW) and treated wastewater (TW) were collected from the wastewater treatment plant (WWTP) with a population equivalent (PE) of 1800. There are no major industrial plants located in the area covered by the WWTP, and therefore mainly domestic wastewater flows to the plant. The lack of industry is reflected in the magnitude of the pollution indicators in the raw wastewater, where a COD/BOD ratio of less than 2.0 was observed. A detailed comparison of pollution indicators in raw and treated wastewater is summarized in Table 2.

The raw wastewater was collected after the processes of mechanical treatment, where wastewater enters an anaerobic chamber in order to eliminate solids from the sample that could be responsible for the processes of sorption or desorption of pollutants in wastewater. The treated wastewater was collected downstream of its outflow from secondary settling tanks, from which the wastewater was collected in the form in which it is delivered to the environment. After collection, the wastewater samples were stored at −20 °C to minimize the activity of chemical and biological processes. To remove solid suspension, the wastewater was filtered with the use of filter paper with a diameter of 150 mm, grammage of 65 g/m^2^ and grade 3; then, it was sterilized for 20 min at a temperature of 121 °C. The sterilized wastewater at room temperature was used for laboratory analyses.

### 2.4. The Inhibition of E. coli Cell Viability Test in MM, RW and TW

For determination of *E. coli* (ATCC 25922) cell viability after treatment with tested chemicals, a BacTiter-Glo™ Microbial Cell Viability Assay Kit (Promega, Madison, WI, USA) was used. In this test, the viability of microbial cells is measured based on the quantification of the ATP present in cells. The stable luminescent signal generated by bacterial cells is proportional to the amount of ATP present in viable cells in the culture. This method is commonly used to test the sensitivity of bacteria to chemical compounds (Promega instructions). The Müeller Hinton II broth (MH II) and raw and treated wastewater were used for cultivation of the *E. coli* ATCC 25922 strain at 37 °C for 24 h. Müeller Hinton II broth (MH II) is an antimicrobial susceptibility testing medium that can be used in internationally recognized standard procedures. It consists of beef extract (300 g per litr), casein hydrolyzate (17, 5 g per litr) and starch (1, 5 g per litr) and the conditions are a pH of 7, 3 at 25 °C. After this, the *E. coli* were diluted in fresh MH II broth and raw and treated wastewater and incubated at 37 °C with shaking (150 rpm) to logarithmic growth phase (OD_600_ = 0.2). FLU, Nor-FLU, NA, CA and their mixtures in the mentioned concentrations were added to the *E. coli* strain culture (10^7^ CFU/mL) and incubated for 24 h. To control samples, no chemical compounds were added. Considering the possible influence of DMSO on *E. coli*, in all experiments, the same volumes of DMSO as in the tested samples were added to the control samples. The assays were performed in accordance with the attached Promega manual. Luminescence values were read on a GloMax^®^-Multi Detection System (Promega) plate reader. In all tests, the luminescence values were normalized to the bacterial concentration, which was measured spectrophotometrically as the OD value at 600 nm. The toxicity of test compounds toward *E. coli* strain was presented as a percent of the inhibition of viability of bacteria cells in comparison to the control. The assays were performed in three independent series.

### 2.5. Toxicity and Protein Damage Effect Determination in MM, RW and TW

Toxicity and protein damage effects of FLU, Nor-FLU, NA, CA and their mixtures in MM, RW and TW were determined with bacterial bioindicator *Escherichia coli* RFM443 strains with reporter plasmids harboring a *grpE* and *lac* promoter sequence fused to the *luxCDABE* reporter genes. *E. coli* strain RFM443, harboring the *pGrpELux* plasmid, is specifically responsive to protein damage and general toxicity. *E. coli* strain RFM443, harboring the *pLacLux* plasmid, generates luminescence constitutively. In this strain, the luminescence decreases when the cells are exposed to toxic chemicals or stresses. These bioindicator strains are based on bioluminescence production resulting from the action of the enzyme luciferase [25]. *Escherichia coli* strains were cultured overnight in Luria Bertani (LB) medium and raw and treated wastewater with 50 μg/mL of ampicillin at 37 °C. Next, the bacteria culture was transferred to fresh LB and regrown at 37 °C with shaking (150 rpm) to the early log phase (OD_600_ = 0.2). Then, in 96-well white plates with optical bottom (Grainer Bio One, Frickenhausen, Germany), 100 μL of appropriate concentrations of the tested chemicals and their mixtures were added to 100 μL of *E. coli* culture (OD_600_ = 0.2). The plates were incubated at 37 °C for 2 and 24 h. Luminescence was measured using a GloMax^®^-Multi Microplate Multimode Reader (Promega) at time zero, after 2 and 24 h of incubation. The luminescence values were normalized by measuring the bacterial concentration using a spectrophotometer at a wavelength of 600 nm. The intensity of luminescence for each tested sample and control was calculated according to the formula: L = IL_A_/OD_600_, where L—luminescence, IL_A_—raw luminescence and OD_600_—optical density of the sample, which was measured spectrophotometrically at 600 nm. The toxic and protein damage effects were presented as a *GrpE* promoter induction value (%) after 2 h of *E. coli* RFM443 *grpE: luxCDABE* culture incubation with test compounds and compared to the control. The experiment was carried out in three replications.

### 2.6. Oxidative Stress Assay in MM, RW and TW

#### 2.6.1. SodA Promoter Induction in MM, RW and TW

The increased levels of free radicals in cells lead to the destruction of important cellular molecules, such as proteins, lipids, DNA and cellular structures. In the course of evolution, cells have developed a number of systems to fight free radicals. One of them is in the form of superoxide dismutase (SOD). SOD carries out the dismutation process of superoxide anion into oxygen and H_2_O_2_ [30]. The state of oxidative stress in the cell induces the *sodA* promoter. The intensity of the *sodA* promoter induction is proportional to the level of superoxide stress in *E. coli* SM342/pBRlux-trp:*sodA:luxCDABE* and the luminescence signal. The strain of bacteria was grown overnight in LB broth, RW and TW at 37 °C. Determination of the level of *sodA* promoter induction and the level of luminescence was performed in accordance with the method described by Melamed et.al. [24] and Kessler et al. [31], with modifications. After that, bacteria culture was diluted in fresh LB, RW and TW and regrown at 37 °C with shaking (150 rpm) to early log phase (OD_600_ = 0.2). Then, 100 microliters of FLU, Nor-FLU, NA solutions and their mixtures were added to 96-well plates with optical bottom (Grainer Bio One, Germany) to 100 microliters of *E. coli* SM342/pBRlux-trp:*sodA:luxCDABE* culture in LB medium and raw and treated wastewater (OD_600_ = 0.2). The samples were incubated at room temperature for 3 h. With the use of GloMax^®^ plate reader (Promega, Walldorf, Germany), the luminescence was measured at time zero and after 3 h of incubation. Simultaneously, the bacterial concentration was monitored spectrophotometrically at 600 nm. The final intensity of luminescence was calculated according to the formula: L = IL_A_/OD_600_, where L—luminescence, IL_A_—raw luminescence and OD_600_—optical density of bacteria culture, which was measured spectrophotometrically at 600 nm. The oxidative stress assay was presented as a *sodA* promoter induction (%) of tested samples in comparison to the control sample. The measurement was performed in three independent series.

#### 2.6.2. ROS Synthesis in MM, RW and TW

The elevated intracellular levels of reactive oxygen species reflect the levels of oxidative stress. Overproduction of ROS causes damage to lipids, proteins and DNA. Bacteria are a very good model object in the study of oxidative stress and for the rapid detection of ROS due to their fast response, high growth rate and low cost [32]. ROS generation in *E. coli* K-12 culture after treatment with FLU, Nor-FLU, NA and their mixtures was determined using a 2′, 7′-dichlorofluorescein diacetate (DCFH-DA) (Sigma-Aldrich, Gillingham, UK) in accordance with the method described by Ong et al. [33] and Díaz-García et al. [34]. DCFH-DA can detect a broad range of ROS, including nitric oxide and hydrogen peroxide. The cultivation of *E. coli* was carried out in LB broth and raw and treated wastewater at 37 °C overnight. After that, bacteria culture was diluted 50-fold in fresh LB broth and raw and treated wastewater and then incubated at 37 °C with shaking (150 rpm) to reach the log phase (OD_600_ = 0.2). Test chemicals and their mixtures used in appropriate concentrations were added to 10^7^ CFU/mL of *E. coli* cultures. Then, DCFH-DA was added to a final concentration of 5 μM and incubated at 37 °C for 50 min. There were no chemicals in the control sample. Measurement of the fluorescence intensity of DCF was performed using GloMax^®^ plate reader (Promega) at an excitation wavelength of 485 nm and the emission wavelength of 535 nm. Simultaneously, with use of a spectrophotometer at 600 nm, the OD values of bacteria cultures were monitored. The ROS level in *E. coli* cultures was presented as a percent of ROS increase comparable to the control sample. All the experiments were performed in triplicate.

### 2.7. Statistical Analysis

The aim of the performed statistical analysis was to determine the nature of the influence of individually tested substances and their mixtures on the induction of the *grpE* promoter, inhibition of bioluminescence in *E. coli lac:luxCDABE*, inhibition of *E. coli* culture viability, cytotoxicity, induction of the *sodA* promoter in *E. coli* SM345/*pBRlux-trp:sodA:luxCDABE* and ROS, depending on the type of culture medium. This type of analysis was carried out with the use of classification methods using the Ward agglomeration algorithm, which makes it possible to indicate all connections occurring in the data set. The effect of agglomeration was the selection of groups of substances or their mixtures characterized by a similar effect to the tested strains. In the case of methods based on agglomeration, it is important that individual variables adopted for the analysis are expressed in convergent units. This allows for limiting the impact of discrepancies in the results of the analysis, which result from uneven numerical values. Therefore, all numerical values included in the classification analysis were expressed as a percentage. In addition, it was verified which of the studied substances differed statistically significantly between the control sample and the studied sample at the α = 0.05 level using Tukey’s test for equal samples.

Next, we determined the effect of individual components of the mixture on the induction of the *grpE* promoter, inhibition of bioluminescence in *E. coli lac:luxCDABE*, the viability of *E. coli* culture inhibition, cytotoxicity, induction of the *sodA* promoter in *E. coli* SM345/*pBRlux-trp:sodA:luxCDABE* and ROS. This analysis was carried out with the use of experimental planning methods for two- and three-valued variables. These variables were represented by the concentration of the individual studied substances, which was 0.01, 0.1 or 1.0 mg/dm^3^. As a result of the implementation of the experimental planning methods, a numerical model was created, which is reflected in the response area. It is the basis for the assessment of the influence of individual components of the mixture on the given measured value. As a result of the numerical analysis, a mathematical model was developed describing the relationship between the concentration of the individual components of a mixture and their influence on the effect of a given toxicity parameter. This makes it possible to identify which component of the mixture contributes most to its effect on the value of the induction of the *grpE* promoter, inhibition of bioluminescence in *E. coli lac:luxCDABE*, inhibition of *E. coli* culture viability, cytotoxicity, induction of the *sodA* promoter in *E. coli* SM345/*pBRlux-trp:sodA:luxCDABE* and ROS depending on the type of culture medium.

## 3. Results

### 3.1. The Inhibition of E. coli Cell Viability in MM, RW and TW

**Figure 1:**
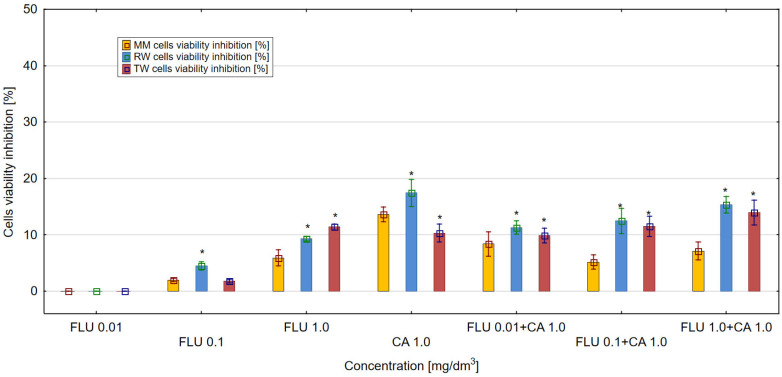
The effect of FLU, CA and their mixtures on *E. coli* (ATCC 25922) cell viability after 24 h of incubation in MM, RW and TW (*—statistically significant difference at α = 0.05). Error bars indicate the standard error of the mean of three independent replicates.

The obtained results showed that FLU, CA and their mixtures influenced the viability of *E. coli* (ATCC 25922) cultures in MM, RW and TW compared to control samples. For FLU, at a concentration of 1 mg/dm^3^, the highest values of the inhibition of bacteria culture viability above 11% were obtained in TW, were lower in RW and were the lowest in MM. In the microbial medium (MH II broth), FLU used at a concentration of 1 mg/dm^3^ inhibited the viability of the *E. coli* culture up to only 5.9% (Figure 1). The highest antibacterial activity above 17% was demonstrated by CA in RW at a concentration of 1 mg/dm^3^. In comparison to the control, the strong toxic effect on *E. coli* cultures was detected only in RW and TW in the mixtures of FLU with CA. The higher antibacterial activity was almost 15% in relation to *E. coli*, as demonstrated in RW for the mixture of FLU at a concentration of 1 mg/dm^3^ with CA at a concentration of 1 mg/dm^3^. Different results were obtained in MM and TW, where the highest values of inhibition of viability of *E. coli* culture, amounting to over 7 and 13%, were observed for mixtures of FLU at a concentration of 1 mg/dm^3^ with CA at a concentration of 1 mg/dm^3^. In MM and RW, comparable to CA, it can be seen that the addition of CA at a concentration of 1 mg/dm^3^ to FLU at a concentration of 1 mg/dm^3^ significantly lowers the toxic potential of the mixtures compared to the total toxicity of their individual components. Considering the mechanisms of toxicity of the tested mixtures, potentiation, as well as antagonistic effects of the mixture components, was detected.

### 3.2. Toxicity and Protein Damage Effect Determination in MM, RW and TW

**Figure 2:**
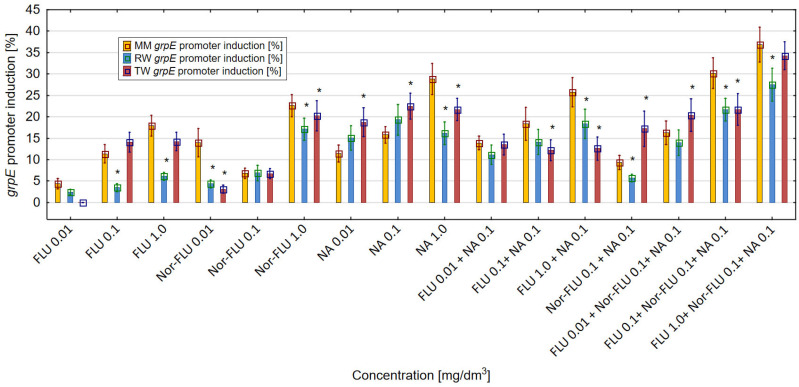
The effect of FLU, Nor-FLU, NA and their mixtures on *grpE* promoter induction in *E. coli grpE:luxCDABE* in MM, RW and TW (*—statistically significant difference at α = 0.05). Error bars indicate the standard error of the mean of three independent replicates.

The induction of the *grpE* promoter is proportional to the toxic effect and the level of protein damage in the *E. coli* RFM443 culture incubated with FLU, Nor-FLU, NA and their mixtures. In MM, RW and TW, both FLU and Nor-FLU, as well as NA and their mixtures, induced the *grpE* promoter as compared to the control sample (Figure 2).

Moreover, on the basis of the obtained results, it was noticed that the type of research environment (MM, RW and TW) had a clear influence on the level of the *grpE* promoter induction. For the tested chemical compounds and their mixtures, the highest values of induction of the *grpE* promoter in *E. coli* culture were detected in MM, were lower in TW and were the lowest in RW. In MM, the metabolite Nor-FLU, especially at concentrations of 0.01 and 1 mg/dm^3^, was much more toxic than the analogous concentration of FLU, and it caused more protein damage in the *E. coli* culture. Among the tested chemicals, the strongest *grpE* promoter induction was detected for NA at a concentration of 1 mg/dm^3^, at over 28%. In MM, in the case of the mixtures, the mixtures containing the three components of FLU with Nor-FLU and NA showed a stronger effect on the *grpE* promoter compared to the mixtures of FLU with NA. The values of the *grpE* promoter induction above 30% were obtained for mixtures of FLU at a concentration of 0.1 and 1 mg/dm^3^ with Nor-FLU at a concentration of 1 mg/dm^3^ and with NA at 1 mg/dm^3^. In MM, in all of the tested mixtures, the dominant toxicity mechanism was the antagonistic effect of the mixture components. A completely different pattern of *grpE* promoter reactivity after its induction by the tested chemical compounds and their mixtures was obtained in RW and TW compared to MM. In RW, at each of the tested concentrations, a stronger toxic effect was observed for the metabolite Nor-FLU compared to FLU. Of the tested chemical compounds, NA at 0.1 mg/dm^3^ induced the strongest, up to over 19%, *grpE* promoter. At the same time, from the tested mixtures, the highest level, at more than 20%, of *grpE* promoter induction was obtained in the mixtures of FLU at 0.1 and 1 mg/dm^3^, with Nor-FLU at 0.1 mg/dm^3^ and NA at 0.1 mg/dm^3^. The main mechanism of toxicity in the mixtures was the antagonistic effect of the mixture ingredients. In TW, Nor-FLU at the highest concentration of 1 mg/dm^3^ showed a higher induction of the *grpE* promoter in the *E. coli* culture compared to the analogous concentration of FLU. In RW, the *grpE* promoter was most intensively affected by NA at 0.1 mg/dm^3^, which caused its induction to increase by more than 20%. Among the mixtures, the highest level of *grpE* promoter induction, at a value of 34.3%, was observed after the incubation of bacteria cultures with the mixture of FLU at 1 mg/dm^3^, with Nor-FLU at 0.1 mg/dm^3^ and NA at 0.1 mg/dm^3^. In mixtures, the main toxicity mechanism was the antagonistic effect of the mixture components.

**Figure 3:**
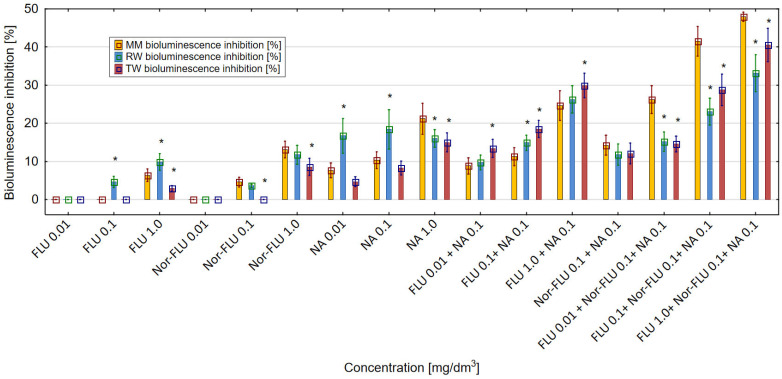
The effect of FLU, Nor-FLU, NA and their mixtures on bioluminescence inhibition in *E. coli lac:luxCDABE* in MM, RW and TW (*—statistically significant difference at α = 0.05). Error bars indicate the standard error of the mean of three independent replicates.

The decrease in luminescence of the *E. coli* RFM443 *lac:luxCDABE* culture was proportional to the cytotoxicity potency of FLU, Nor-FLU, NA, CA and their mixtures. All of the tested chemical compounds, in various concentrations and their mixtures, showed a cytotoxic effect in the tested bacterial culture. Regardless of the tested environment (MM, RW and TW), FLU and Nor-FLU induced the highest level of luminescence inhibition in the bacterial culture at the highest concentration tested (Figure 3). Nor-FLU, especially at the highest concentration of 1 mg/dm^3^, showed a higher degree of inhibition of luminescence in the bacterial culture compared to the corresponding concentration of FLU. The obtained results showed that the type of research environment (MM, RW and TW) influenced the cytotoxic activity of the tested chemical compounds and their mixtures. Of the tested chemical compounds, NA at 1 mg/dm^3^ showed the strongest cytotoxic activity, up to over 21%, in the *E. coli* RFM443 culture. In MM mixtures of FLU with NA, there was a stronger effect on the luminescence of the bacteria cultures compared to these mixtures in RW and TW. In the three tested environments (MM, RW and TW), the highest values of luminescence inhibition, even up to over 40% in MM, were obtained for the mixture of FLU at 1 mg/dm^3^, Nor-FLU at 0.1 mg/dm^3^ and NA at 0.1 mg/dm^3^. In RW and TW, these values were lower. Regarding the mechanism of cytotoxicity, we distinguish potentiation and the synergistic and antagonistic actions of the mixture components.

The enhancement of the toxicity in the mixtures as compared to the total toxic effect of the individual components of the mixtures was found in the following cases: FLU at 1 mg/dm^3^ with NA at 0.1 mg/dm^3^ (in MM), FLU at 0.01, 0.1 and 1 mg/dm^3^ with Nor-FLU at 0.1 mg/dm^3^ and with NA at 0.1 mg/dm^3^ (in MM) and FLU at 1 mg/dm^3^ and with NA at 0.1 mg/dm^3^ (in TW). In the case of these mixtures, the mechanism of their toxicity was the synergistic action of their components.

**Figure 4:**
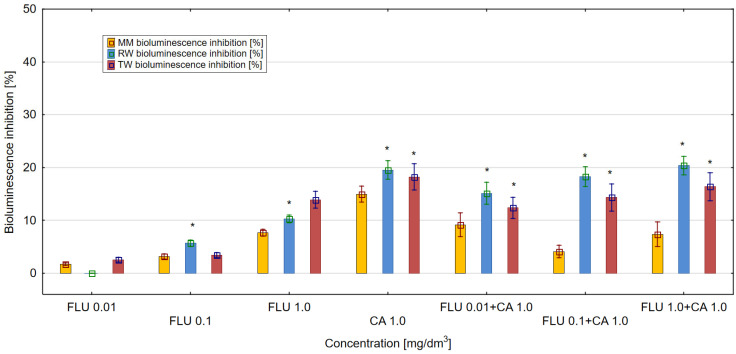
The effect of FLU, CA and their mixtures on bioluminescence inhibition in *E. coli lac:luxCDABE* in MM, RW and TW (*—statistically significant difference at α = 0.05). Error bars indicate the standard error of the mean of three independent replicates.

On the other hand, CA at 1 mg/dm^3^ in the three tested environments (MM, RW and TW) showed a stronger toxic effect compared to FLU tested at the same concentration (Figure 4). In the case of mixtures of FLU with CA, the strongest toxic effect in the culture of *E. coli* RFM443 *lac:luxCDABE* of more than 20% was detected in RW for the mixture of FLU at a concentration of 1 mg/dm^3^ with CA at 1 mg/dm^3^. In MM and TW, the toxicity values of the mixtures were lower. The mechanisms of toxicity of the tested mixtures were dominated by the antagonistic action of their components.

### 3.3. Oxidative Stress Assay in MM, RW and TW

#### SodA Promoter Induction in MM, RW and TW

**Figure 5:**
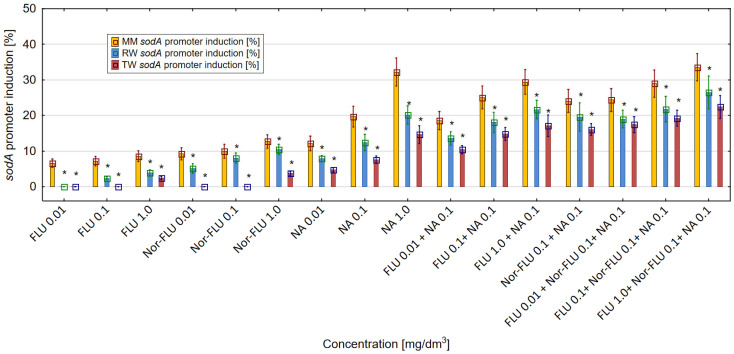
The effect of FLU, Nor-FLU, NA and their mixtures on *sodA* promoter induction in *E. coli sodA:luxCDABE* in MM, RW and TW (*—statistically significant difference at α = 0.05). Error bars indicate the standard error of the mean of three independent replicates.

The oxidative stress level was proportional to the intensity of *sodA* promoter induction and luminescence generation in the *E. coli* SM345 *sodA*:*luxCDABE* strain. Results obtained in the research showed that all of the tested chemicals and their mixtures influenced the activity of the *sodA* promoter in *E. coli* SM345 *sodA*:*luxCDABE* (Figure 5). For the analyzed compounds and their mixtures, the highest level of *sodA* promoter induction was detected in MM, was lower in RW and was the lowest in TW. In MM, the greatest differences were observed between the *sodA* promoter induction by FLU and its metabolite Nor-FLU. From the analyzed compounds and regardless of the studied environment (MM, RW and TW), the highest level of induction of the *sodA* promoter above 30% in MM was detected at the highest concentration of NA at 1 mg/dm^3^. The mixtures of FLU at 1 mg/dm^3^ with NA at 0.1 mg/dm^3^ and FLU at 1 mg/dm^3^ with Nor-FLU at 0.1 mg/dm^3^ and with NA at 0.1 mg/dm^3^ showed the highest induction of the *sodA* promoter above 33% among the tested mixtures in MM, RW and TW. In the tested mixtures, the main mechanisms of the *sodA* promoter induction were potentiation, normal additivity and the antagonistic and synergistic action of the mixture components.

The enhancement of the induction of the *sodA* promoter in the mixtures as compared to the total *sodA* induction of the individual components of the mixtures was found in the following cases: FLU at 0.1 and 1 mg/dm^3^ with NA at 0.1 mg/dm^3^ (in RW), FLU at 1 mg/dm^3^ with NA at 0.1 mg/dm^3^ (in TW), FLU at 1 mg/dm^3^ with Nor-FLU at 0.1 mg/dm^3^ and with NA at 0.1 mg/dm^3^ (in TW). In the case of these mixtures, the mechanism of *sodA* induction was the synergistic action of their components.

### 3.4. ROS Generation in MM, RW and TW

**Figure 6:**
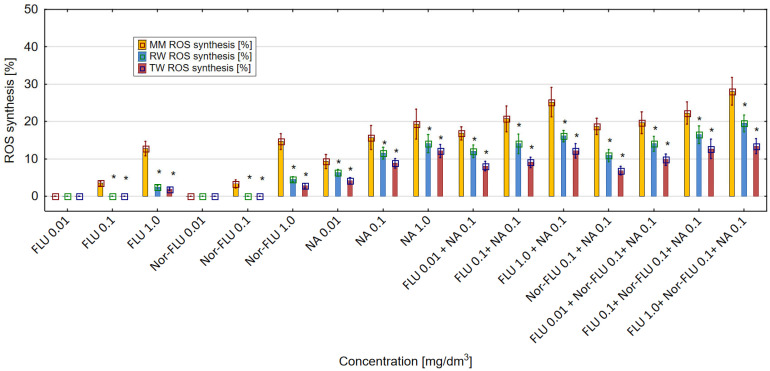
The effect of FLU, Nor-FLU, NA and their mixtures on ROS synthesis in *E. coli* (ATCC 25922) in MM, RW and TW (*—statistically significant difference at α = 0.05). Error bars indicate the standard error of the mean of three independent replicates.

The obtained results showed that FLU, Nor-FLU, NA and their mixtures triggered ROS synthesis in the *E. coli* (ATCC 25922) culture (Figure 6). For all the tested chemical compounds and their mixtures, the strongest generation of ROS was detected in MM, was lower in RW and was the lowest in TW. In MM, RW and TW the metabolite Nor-FLU tested at a concentration of 1 mg/dm^3^ showed a similar ROS generation potential compared to the analogous concentration of FLU. Regardless of the tested environment, NA at a concentration of 1 mg/dm^3^ showed the strongest ROS generation activity, above 19% in MM, comparable to the control sample. From the tested mixtures, the highest potential for ROS synthesis, over 25 and 28%, was found in MM and for the mixtures of FLU at 1 mg/dm^3^ with NA at 0.1 mg/dm^3^ and FLU at 1 mg/dm^3^ with Nor-FLU at 0.1 mg/dm^3^ and with NA at 0.1 mg/dm^3^. In the case of the tested mixtures, the mechanisms of ROS synthesis were potentiation and normal additivity, as well as the synergistic and antagonistic actions of the mixture components.

The enhancement of ROS synthesis in the mixtures as compared to the total ROS synthesis of the individual components of the mixtures was found in the following cases: FLU at 1 mg/dm^3^ with NA at 0.1 mg/dm^3^ (in RW), FLU at 1 mg/dm^3^ with NA at 0.1 mg/dm^3^ (in TW), FLU at 1 mg/dm^3^ with Nor-FLU at 0.1 mg/dm^3^ and with NA at 0.1 mg/dm^3^ (in RW) and FLU at 0.1 mg/dm^3^ with Nor-FLU at 0.1 mg/dm^3^ and with NA at 0.1 mg/dm^3^ (in TW). For these mixtures, the mechanism of ROS synthesis was the synergistic action of their components.

### 3.5. Statistical Analysis

The discussed results of the research were reflected in the analysis of agglomeration carried out by Ward’s method, with the help of which groups of mixtures showing a similar effect on the tested parameters in individual types of matrices were identified. The obtained results indicated that the type of culture medium directly translated into the toxicity of single compounds or their mixtures. In the case of the culture medium, it was observed that a clear increase in toxicity was caused by three-component mixtures, while two-component mixtures and single compounds showed a similar nature of the effect on all the studied parameters. Taking into consideration the values presented in Figure 1, Figure 2, Figure 3, Figure 4, Figure 5 and Figure 6, it can be concluded that this regularity is dictated by the fact that, in the case of three-component mixtures, the greatest increase in the values of the individual studied parameters was observed, which in turn corresponds to an increase in toxicity. Hence, using agglomeration methods, it was shown that three-component mixtures of the studied substances have greater toxicity compared to two-component mixtures and single substances.

**Figure 7:**
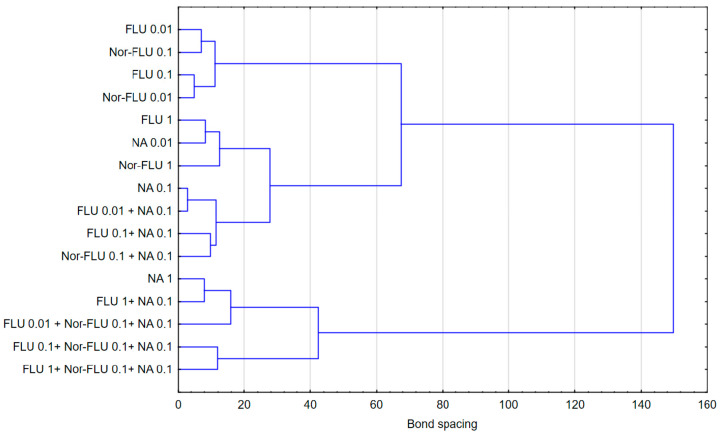
Similarity of the action of FLU, Nor-FLU, NA and their mixtures on *grpE*, *lacZ*, *sodA* promoters and ROS synthesis in MM.

**Figure 8:**
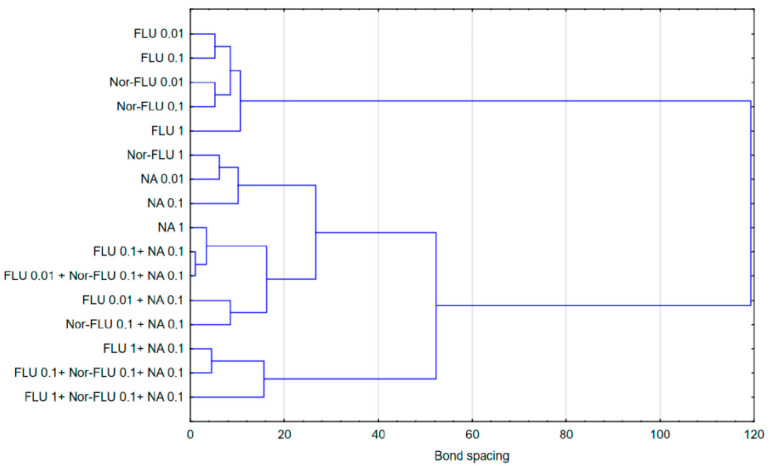
Similarity of the action of FLU, Nor-FLU, NA and their mixtures on *grpE*, *lacZ*, *sodA* promoters and ROS synthesis in RW.

In the case of raw and treated wastewater, on the other hand, it was observed that single compounds had a different effect compared to two-component and three-component mixtures. Referring to the results presented in Figure 1, Figure 2, Figure 3, Figure 4, Figure 5 and Figure 6, this observation indicates that the probable presence of organic and mineral micropollutants may contribute to the increased toxicity of two-component mixtures of the studied pharmaceuticals. The Ward’s agglomeration results obtained for the culture medium are shown in Figure 7 and for the raw wastewater are in Figure 8.

**Figure 9:**
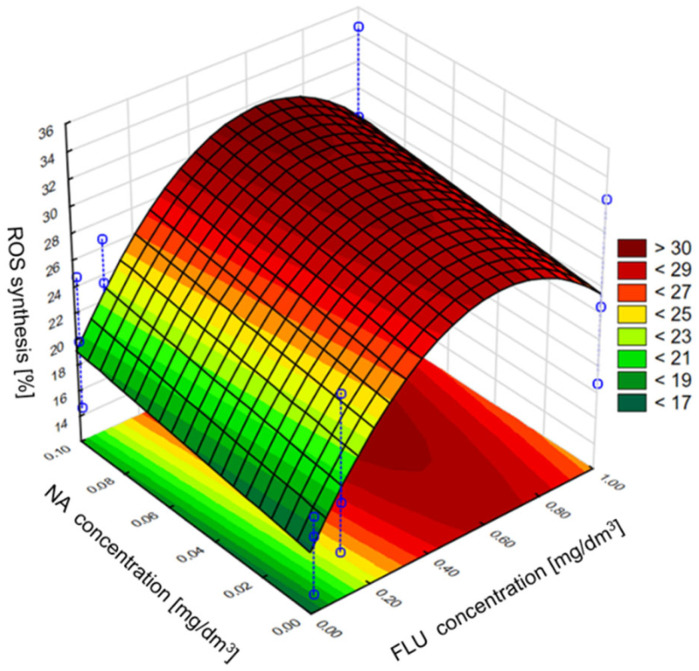
The effect of individual components of the mixture of FLU with NA on ROS synthesis in *E. coli* in MM.

**Figure 10:**
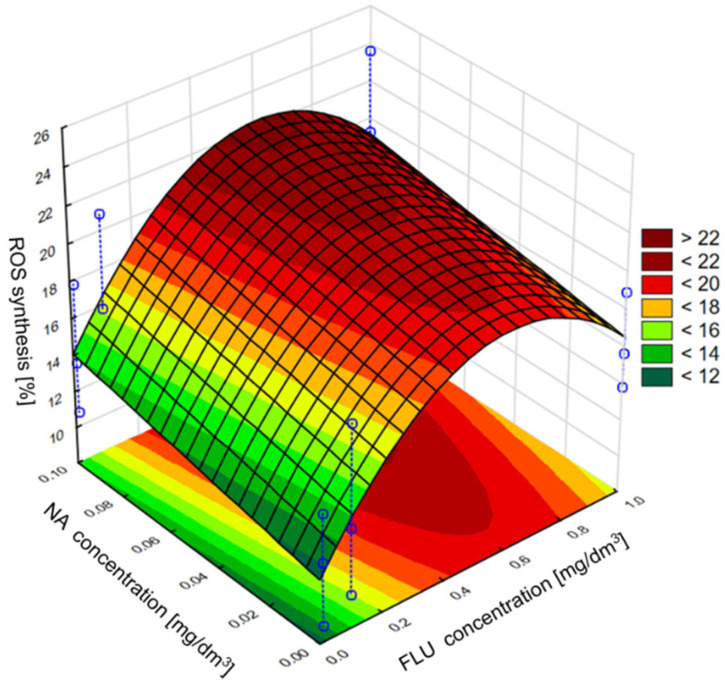
The effect of individual components of the mixture of FLU with NA on ROS synthesis in *E. coli* in RW.

Next in the statistical analysis, we assessed the effect of individual components of the mixture on the induction of the *grpE* promoter, inhibition of bioluminescence, induction of the *sodA* promoter and ROS synthesis. In the case of the *grpE* promoter induction, it was observed that, in the culture medium and raw wastewater, the influence of the mixture on the induction was determined only by the tested concentration of FLU, while the addition of NA in the analyzed concentrations may have contributed to the intensification of FLU activity. It was also found that the highest of the tested concentrations of FLU did not show the highest induction of the *grpE* promoter, which, according to the developed model, occurs at the concentration of FLU at about 0.6 mg/dm^3^ and NA at 1 mg/dm^3^. On the other hand, in TW, the effect of NA on the toxicity of FLU was clearly noticed, where the greatest effect of mixtures containing these compounds was observed at the highest concentrations of FLU and NA tested. In the case of bioluminescence inhibition, it was observed that, in the culture medium, NA catalyzes the effect of FLU on the value of this indicator, and the most intense influence on this parameter was detected at the highest concentrations of FLU and NA. However, in raw and treated wastewater, the concentration of FLU itself turned out to be more important, as it clearly influenced the observed inhibition of bioluminescence with its increase, while the presence of NA had a slight influence on the toxicity of the whole mixture. In the case of induction of the *sodA* promoter and the synthesis of ROS in the culture medium, a clear influence of FLU on the values of the discussed parameters was observed. The presence of NA in the mixture did not significantly influence its toxicity. However, with the change in the type of research environment (MM, RW and TW), the addition of NA clearly influenced the effect of FLU in terms of *sodA* promoter induction and ROS synthesis, contributing to the reduction in the toxic effect of FLU compared to the culture medium. Examples of the developed models are shown in Figure 9 and Figure 10, which refer to the mixture of FLU and NA on ROS in the culture medium and raw wastewater, respectively.

## 4. Discussion

The results obtained in this paper indicate that FLU, Nor-FLU, NA and their mixtures show toxicity and have the potential to damage cellular proteins in *E. coli* RFM443 *grpE:luxCDABE* and *E. coli* RFM443 *lac:luxCDABE* cultures in MM, RW and TW. Previous work has documented the antimicrobial activity of SSRIs, including FLU, against some Gram-positive bacteria. These drugs were also active against other microorganisms, such as *Haemophilus influenzae*, *Moraxella catarrhalis*, *Campylobacter jejuni* and *Acinetobacter*, some microorganisms belonging to *Staphylococci* and *Enterococci*, and anaerobes of the species *Bacteroides fragilis* [35,36,37]. The results of our research presented in this study showed that, in MM, RW and TW, the highest values of toxicity were detected in mixtures of FLU at concentrations of 0.1 and 1 mg/dm^3^ with Nor-FLU (0.1 mg/dm^3^) and with NA (0.1 mg/dm^3^) and FLU (1 mg/dm^3^) with NA (0.1 mg/dm^3^). It was also found that, in the case of these mixtures, mainly in MM, their toxicity was higher in the *E. coli* cultures compared to the total effect of the toxicity of the individual components of the mixtures. Similarly, in the works of Karine de Sousa et al. [8], the authors found antibacterial activity of FLU against standard and multiresistant strains of *Pseudomonas aeruginosa*, *Staphylococcus aureus* and *Escherichia coli*. In addition, in laboratory experiments carried out by Foletto et al. [38], significant antimicrobial activity of FLU was detected against strains *Acinetobacter baumannii* MDR and *Escherichia coli* ATCC 35218. Karine de Sousa et al. [8] also documented the effect of enhancing the antimicrobial activity of FLU in mixtures with known antibiotics: erythromycin, gentamicin, imipenem, norfloxacin and tetracycline. In the conducted research, the authors found significant antibacterial activity of FLU against the test bacteria strains. An unfavorable effect of FLU was also observed, which inhibited the proper development of *Sepia officinalis* exposed to this drug at a concentration of up to 1 ng/dm^3^. It was also noted that FLU at concentrations of 1–100 ng/dm^3^ had an adverse effect on the swimming and feeding processes of *Gammarus pulex*, and at a concentration of 20 ng/dm^3^, it disrupted the endocrine system in the mussel *Dreissena polymorpha* [6,10].

In the presented research, we obtained strong toxic activity of NA and its mixtures with FLU and Nor-FLU. NA is an antibiotic belonging to the quinolone group. NA exhibits antimicrobial properties against Gram-negative bacteria, including *Enterobacter* species, *Escherichia coli*, *Morganella morganii*, *Proteus mirabilis*, *Proteus vulgaris* and *Providencia rettgeri*. Moreover, NA showed cytotoxic and anticancer activities [39].

In the human body, FLU undergoes demethylation, resulting in its metabolite, Nor-FLU. In our research, we found that the metabolite Nor-FLU, mainly at the concentration of 1 mg/dm^3^ in the three tested environments, showed higher toxicity compared to the analogous concentration of FLU. The obtained results are consistent with the results of the work of López-Muñoz and Alamo [40], who proved in tests on mice and rats that some of the metabolites of antidepressants are highly toxic. In addition, Tiwari et al. [41], in their work, reported that the metabolite Nor-FLU may be more toxic in comparison to the parent drug FLU.

In the presented work, *E. coli* strains containing gene constructs with two different promoters, *grpE* and *lac*, were used to determine the toxicity of FLU, Nor-FLU, NA and their mixtures in MM, RW and TW. Our results showed that a higher level of toxicity, especially in the mixtures of FLU with NA and FLU with Nor-FLU and with NA was achieved in the case of the *lac* promoter. Using this promoter, it was also possible to detect the enhancement of the toxicity of mixtures, especially the mixture of FLU with Nor-FLU and with NA, compared to the general toxicity of their individual components. This proves that the *lac:luxCDABE* gene construct is more sensitive in determining the toxicity of tested compounds and their mixtures compared to the *grpE:luxCDABE* construct.

In our studies, we discovered that CA at a concentration of 1 mg/dm^3^ and its mixtures with FLU are toxic to *E. coli* strains. The antimicrobial activity of CA against Gram-positive and Gram-negative bacteria and *C. albicans* was documented in earlier works. The biological mechanism is probably associated with one more hydroxyl group substituted at the CA phenol ring [42]. Similarly, antimicrobial properties were observed in FLU. Due to the fact that both FLU and CA show antibacterial properties, they may influence the microflora of the activated sludge in WWTPs. CA at higher doses of mg/kg and g/L is also toxic to higher organisms such as mice, rats and humans [19].

In some scientific studies, it has been documented that chemicals that pollute the environment, including drugs, cause oxidative stress. In many cases, oxidative stress is a mechanism for the toxicity of environmental pollutants [43]. Microbiological bioindicators with the *sodA* promoter and the *luxCDABE* reporter gene have been used to assess the level of oxidative stress in living cells [24,26,27,28]. In our research described in this paper, it was noticed that in MM, RW and TW, FLU, Nor-FLU and NA and their mixtures had an effect on the activity of the *sodA* promoter and the level of *luxCDABE* gene expression in *E. coli* SM345 *sodA: luxCDABE*. The strongest effect of induction of the *sodA* promoter in the culture of *E. coli* SM345 *sodA*: *luxCDABE* was noticed after its exposition to NA at the highest tested concentration of 1 mg/dm^3^ and the mixture of FLU (0.01, 0.1 and 1 mg/dm^3^) with Nor-FLU (0.1 mg/dm^3^) and NA (0.1 mg/dm^3^) in MM. It was also found that mixtures of FLU (0.1 and 1 mg/dm^3^) with NA (0.1 mg/dm^3^) in MM and TW and FLU (1 mg/dm^3^) with Nor-FLU (0.1 mg/dm^3^) and NA (0.1 mg/dm^3^) in TW showed a higher induction of the *sodA* promoter as compared to the total induction of this promoter by individual components of the mixtures. Moreover, all the chemicals and their mixtures analyzed in this work increased the synthesis of ROS. In MM, RW and TW, the highest level of ROS generation was detected in the mixture of FLU (1 mg/dm^3^) with Nor-FLU (0.1 mg/dm^3^) and NA (0.1 mg/dm^3^). It was also noticed that mixtures of FLU (0.1 and 1 mg/dm^3^) with NA (0.1 mg/dm^3^) in RW and TW and FLU (0.1 mg/dm^3^) with Nor-FLU (0.1 mg/dm^3^) and NA (0.1 mg/dm^3^) in TW presented a higher level of ROS generation in comparison to the total ROS synthesis by individual components of the mixtures. The results obtained in our study indicate that oxidative stress may be one of the main toxicity mechanisms of FLU, Nor-FLU, NA and their mixtures. Our results are in agreement with those of other authors, where it was noticed that SSRIs, including FLU, have a toxic and genotoxic effect and have the potential to generate oxidative stress as the main mechanism, leading to the permanent damage of cells exposed to these drugs [44]. The potential for generating oxidative stress in cells exposed to FLU has also been described in other works. Orozco-Hernández et al. [1] conducted an experiment using *D. rerio* embryos, which were exposed to environmentally relevant concentrations of FLU. These studies showed that FLU induces oxidative stress in *D. rerio* tissues and is teratogenic. The authors concluded that FLU is a dangerous drug in the early life stages of *D. rerio* due to its high teratogenic potential and that FLU-induced oxidative stress may be involved in this toxic process. In another work, it was shown that FLU used at a concentration of 500 ng/dm^3^ caused oxidative stress and was genotoxic [10]. On the other hand, in an experiment on mussels of the species *M. galloprovincialis*, it was documented that, under the influence of FLU applied at a concentration of 0.03–300 ng/dm^3^, lipid peroxidation intensification and modulation of the expression of genes involved in the detoxification of xenobiotics occurred [10,45,46].

The scientific literature clearly lacks studies on the impact of wastewater on the toxic activity of drugs, metabolites and their mixtures. In this study, we documented that RW and TW, compared to MH, have a significant effect on the toxicity and the potential of oxidative stress generation in *E. coli* cultures exposed to tested chemical compounds and their mixtures. Earlier studies [47,48,49] showed that the composition of wastewater, the amount of organic matter, the presence of chemicals and the composition of the microflora have an impact on the biological activity and biodegradation efficiency of pharmaceuticals and other chemicals found in wastewater. It should be emphasized that, in the studies presented in this paper, due to the test procedures used, both raw and treated wastewater samples were filtered and autoclaved, which undoubtedly changed their composition and could have had an impact on reducing their toxicity.

## 5. Conclusions

The presented research concerned the estimation of the toxicity and oxidative stress generation of the antidepressant FLU, its metabolite Nor-FLU, the antibiotic NA and CA—a compound of natural origin—and their mixtures in three different environments: MM, RW and TW. The listed drugs and metabolites are detected in the environment. Pharmaceutical toxicity studies have so far mainly been carried out on drug solutions used individually and most often in laboratory microbiological media. In the literature, there are no studies on the toxicity of drugs and their mixtures in wastewater. The current state of knowledge was supplemented by the results of our research presented in this work, and the following conclusions were formulated on this basis:

(1) FLU, Nor-FLU, NA, CA and their mixtures in MM, RW and TW and with varying intensities affect the expression level of the *luxCDABE* reporter gene in *E. coli* strains.
(2) Wastewater significantly influenced the toxicity and oxidative stress generation in *E. coli* cultures exposed to the tested chemical compounds and their mixtures.
(3) The metabolite Nor-FLU showed higher toxicity compared to the parent drug FLU and a slightly higher oxidative stress synthesis.
(4) Regardless of the tested environment, NA showed higher toxicity and ROS synthesis as compared to FLU and Nor-FLU.
(5) Regardless of the tested environment, CA showed higher toxicity compared to FLU.
(6) Among the tested mixtures, the highest toxicity and level of oxidative stress generation were characterized by those containing FLU at a concentration of 1 mg/dm^3^. The most toxic mixture was FLU (1 mg/dm^3^) with Nor-FLU (0.1 mg/dm^3^) and NA (0.1 mg/dm^3^).
(7) An assessment of the interactions between the components of the mixtures in wastewater revealed synergism, potentiation and antagonism.
(8) The *E. coli* microbial biosensors with *grpE*, *lac* and *sodA* promoters and the *luxCDABE* reporter gene used in the research are useful tools in studies on the toxicity and oxidative stress generated by the analyzed chemical compounds and their mixtures in wastewater.

The studies indicate the possibility of various types of interactions between pharmaceuticals and their metabolites in wastewater. The synergistic effect of mixture components may lead to an increase in their toxicity and a stronger toxic impact on the activated sludge. This leads to a disruption in the proper functioning of activated sludge and a lowering of the efficiency of the biodegradation of chemicals in wastewater. This suggests the need to treat wastewater that is particularly loaded with pharmaceuticals, such as hospital wastewater and wastewater from drug production plants. Another task is to reduce the transport of pharmaceuticals into the environment and improve the technology of their disposal in wastewater. Biofilm-based methods and AOPs are currently of great interest when designing wastewater treatment systems containing pharmaceuticals.

It was found that *E. coli* strains with the plasmid-based genetic constructs *grpE:luxCDABE*, *lac:luxCDABE* and *sodA:luxCDABE* are useful as microbial biosensors in the assessment of the impact of the RW and TW environments on the toxicity of FLU, Nor-FLU, NA, CA and their mixtures and oxidative stress generation. The obtained results can be the basis for a wider validation of strains as biosensors for toxicity assessments of micropollutants in water and wastewater.

## Figures and Tables

**Table 1:** *E. coli* bioindicator strains used in this study.

Strain	Gene Promoter Acting as Sensing Element	Reporter Gene	Type of Stress Sensed	Reference
*E. coli* RFM443	*GrpE*	*Lux*	Protein damage and general toxicity	[25]
*E. coli* RFM443	*Lac*	*Lux*	General toxicity	[25]
*E. coli* SM345	*SodA*	*Lux*	Oxidative stress	[24,26,27,28]

**Table 2:** Characteristics of raw and treated wastewater.

Parameter	Raw Wastewater	Treated Wastewater
BOD	250 ± 100	3 ± 2
COD	450 ± 120	30 ± 10
TSS	250 ± 50	4 ± 3

## Data Availability

The data presented in this study are available upon request from the corresponding author.
